# Potency testing of cell and gene therapy products

**DOI:** 10.3389/fmed.2023.1190016

**Published:** 2023-05-05

**Authors:** Paula Salmikangas, Björn Carlsson, Christophe Klumb, Tatiana Reimer, Steffen Thirstrup

**Affiliations:** ^1^NDA Group, Uplands Väsby, Sweden; ^2^European Medicines Agency, Amsterdam, Netherlands

**Keywords:** ATMP, biological activity, potency, cell therapy, gene therapy

## Abstract

Potency is one of the critical quality attributes of biological medicinal products, defining their biological activity. Potency testing is expected to reflect the Mechanism of Action (MoA) of the medicinal product and ideally the results should correlate with the clinical response. Multiple assay formats may be used, both *in vitro* assays and *in vivo* models, however, for timely release of the products for clinical studies or for commercial use, quantitative, validated *in vitro* assays are necessary. Robust potency assays are fundamental also for comparability studies, process validation and for stability testing. Cell and Gene Therapy Products (CGTs, also called Advanced Therapy Medicinal Products, ATMPs) are part of biological medicines, having nucleic acids, viral vectors, viable cells and tissues as starting material. For such complex products potency testing is often challenging and may require a combination of methods to address multiple functional mechanisms of the product. For cells, viability and cell phenotype are important attributes but alone will not be sufficient to address potency. Furthermore, if the cells are transduced with a viral vector, potency probably is related to the expression of the transgene but will also be dependent on the target cells and transduction efficiency/copy number of the transgene in the cells. Genome Editing (GE) together with other cell manipulations can result into multiple changes in the characteristics and activity of the cells, which should be all somehow captured by the potency testing. Non-clinical studies/models may provide valuable support for potency testing, especially for comparability testing. However, sometimes lack of suitable potency data may lead to situations where bridging clinical efficacy data are required to solve the problems of the potency testing, for example where comparability of different clinical batches is unclear. In this article the challenges of potency testing are discussed together with examples of assays used for different CGTs/ATMPs and the available guidance addressing differences between the European Union and the United States.

## Introduction

Biological activity, also called potency, is the critical quality attribute that separates biological medicinal products from those based on small molecules. According to international guidance (1), potency is the quantitative measure of biological activity, which is linked to the relevant biological properties of the product. The assay(s) utilized for potency measurement should be based on the intended biological effect, which ideally should be related to the clinical response. *In vivo* models used during pharmacodynamic (PD), pharmacokinetic (PK) and proof-of-concept (PoC) studies usually provide an early readout of potency by measuring the physiological response in animals. However, for release of medicinal products for clinical studies and for commercial use, *in vitro* assays are required to provide timely and quantifiable outcomes that can be validated.

Advanced Therapy Medicinal Products (2) (ATMPs, including cell and gene therapy medicinal products and tissue engineered products) are part of biological medicines, having nucleic acids, viral vectors, viable cells and tissues as starting material. Considering the diverse and complicated nature of ATMPs their potency testing may require combination of multiple, often novel methods to address all functional mechanisms of the active substance. For cells, viability and cell phenotype are important attributes but alone will not be sufficient to address biological activity. Furthermore, if the cells are transduced with a viral vector, potency probably is related to the expression of the transgene but will also be dependent on the target cells and transduction efficiency/copy number of the transgene in the cells. In principle, a quantitative, functional potency assay is expected for ATMPs (3–7) and the testing should follow the Mechanism of Action (MoA) of the active substance.

According to International Council for Harmonisation (ICH) Q6B guideline (1) potency assays may be 1) animal-based *in vivo* biological assays, 2) cell-based biological assays, 3) biochemical assays, which measure biological activities of the product or 4) other procedures such as ligand and receptor binding assays. In practice for ATMPs the animal-based *in vivo* assays may not be feasible due to the time they take and the difficulties in validating animal-based methods. Mostly used functional assays for ATMPs are cell-based and/or biochemical methods. Depending on the quality control strategy of a given product, the regulators in the EU may allow to use surrogate assay(s) for release testing when a functional assay is available for characterisation and correlation of these assays can be demonstrated (1, 5). This could be the case especially when the functional assay is based on multiple functional mechanisms of the active substance and might be difficult to validate according to ICH requirements (8). US FDA, on the other hand, is expecting a quantitative functional potency assay for release, also for ATMPs (3). Absolute quantification may not be achievable for all ATMPs and a relative potency approach, comparing a test item to a reference standard, could be applied (9, 10).

Potency assays are required for multiple purposes during product development. In addition to release testing of the product, potency assays may be needed to control the product during storage. Thus, the assay(s) should be stability-indicating and able to differentiate between target and degraded product. Process and material changes during production require demonstration of process and product comparability before and after the changes (11). There, robust and reliable potency assays are of outmost importance. Sometimes non-clinical proof-of concept models, if considered relevant, may be useful to support such comparability studies in case information on retained *in vivo* biological responses is required.

## Regulatory expectations for potency testing

Testing for biological activity/potency is expected for all biological medicinal products, including ATMPs. Many jurisdictions have set this as a legal requirement, as for example in section 3.2.2.1. of Part I of the Annex I to the EU Directive 2001/83/EC (12). The US Federal Regulation 21 CFR Part 600.3 (13) states “the word potency is interpreted to mean the specific ability or capacity of the product, as indicated by appropriate laboratory tests or by adequately controlled clinical data obtained through the administration of the product in the manner intended, to effect a given result.” While the US FDA has specific guidance available for potency testing of Cell and Gene Therapy Products (3), in the EU the high-level potency guidance can be extracted from the overarching guidelines for Human Cell-based Guideline (5) and the Guideline on the Quality, Non-clinical and Clinical aspects of Gene Therapy Medicinal Products (6). In addition, specific guidance on potency testing of products intended for cancer immunotherapy (4) and genetically modified cells (7) is available in the EU. Recently, US FDA has issued two new guidelines, one for Chimeric Antigen Receptor T-cells (CAR Ts) (14) and one for Genome Editing (GE) (15); both guidelines address also potency testing of such products.

The regulatory expectations for potency tests of ATMPs vary depending on the phase of development. Validated assays must be in place for commercial production and most guidelines recommend those to be used already for testing of the product before pivotal clinical studies, so that correlation of the potency test results with clinical efficacy can be explored. ATMPs are complex medicinal products that often have multiple mechanisms, by which the intended effect is achieved. For example, CAR T cells are produced by transducing T cells with a viral vector, which allows the CAR construct to be integrated. Thus, the ultimate biological activity is dependent on cell viability, number of vector copies inside the cells, expression of the CAR transgene and the final activity (cytotoxicity) of modified T-cells. For Gene Therapy Products based on viral vectors, correct packaging may impact potency, like in case of Adeno-associated Viruses (AAV) that may contain “empty” particles containing no or only part of the genomic sequence inside the capsid (16). Therefore, all the aspects related to potency testing should be recognized early on and the respective analytical methods developed and qualified side by side (17).

The EMA and FDA guidelines recommend to evaluate multiple potency assays for ATMPs during early development. This is due to the fact that many functional assays may turn out to be difficult to validate or some assays, especially cell-based, may bear high intrinsic variability that can hamper the use of such assays for release or comparability testing. The US FDA requirement to have a functional potency assay for release of ATMPs (3) has caused challenges for some companies developing ATMPs like in case of Lifileucel, intended for cancer therapy (18). The respective EU guidances (5–7) do acknowledge the challenges of functional assays and propose that for release testing validated surrogate assays could be utilized, provided that a functional assay is available as a characterisation tool and that the assays correlate with each other. Analytical methods used for characterisation do not need to be validated according to ICHQ2 (R1) but have to be qualified to ensure their reliability. Validated methods are expected for release and stability testing and testing of in process controls (consistency of the process). Again, this applies mainly to commercial and pivotal clinical batches. For early clinical development, qualified methods are generally accepted (19, 20). However, when the methods change during the development, analytical bridging would be expected as part of comparability studies. This could be done, e.g., by analyzing “split” samples with the old and new method or by analyzing also retained samples using the new method. This, however, would require that samples from produced batches are stored under controlled conditions for later use. This is highly recommended anyway, as unforeseen challenges with potency testing may emerge as late as during the review process of a Marketing Authorization Application (MAA), as described by Barkholt et al. (21). According to the experience of the authors, major issues with potency tests were noted in almost 50% of all ATMP MAAs in the EU.

## Analytical methods

The analytical methods needed for potency testing are specific to the product type and the clinical indication. Plasmids and nucleic acids (e.g., mRNA, siRNA) are close to common biologicals like therapeutic proteins, as those are structurally quite simple and have limited amount of quality attributes that can be controlled. Even viral vectors, although complex, are structurally well defined. Cells, on the other hand, are viable organisms with own organelles involved in protein production, signaling, metabolic/respiratory activity, etc. For cells the amount of possible quality attributes is so vast, possibly hundreds of thousands, that full control of them is impossible. For both cell and gene therapy products the characterisation of the intended active substance should identify those product characteristics and cellular processes that need to be controlled for potency.

### *In vivo* gene therapy products

Gene therapy products include viral vectors, plasmids and nucleic acids administered directly (*in vivo*) to patients, but also *ex vivo* genetically modified cells (addressed under cell-based ATMPs). Most widely used *in vivo* viral products are AAVs and oncolytic viruses like Adenoviruses (AdV) and Herpes Simplex Virus (HSV). Gamma-retro- and Lentiviruses are mainly used for *ex vivo* gene therapy but could be used also for *in vivo* administration (22). Viral vectors can enter target cells through infection, but for non-viral products specific administration approaches are required, e.g., lipid nanoparticles (LNPs) for delivery of nucleic acids (23).

Recombinant AAVs and plasmids usually carry a transgene to express therapeutic protein(s) in the target cells. Potency testing of such vectors and plasmids is focused on assessing the expression level of the protein using suitable cells together with SDS-PAGE (Sodium Dodecyl Sulfate Polyacrylamide Gel Electrophoresis) and Western Blot (WB). Alternatively, protein expression could be detected by histological staining of the cells or by flow cytometry. For all these approaches, a specific antibody against the expressed protein is required. One challenge is that the potency assay should be quantitative; this is possible with the WB, but more difficult with a histological staining. On the other hand, there could be specific functional assays available for the expressed proteins, like in case of enzymes for which enzymatic activity could be measured. Such examples are lipoprotein lipase and retinoid isomerohydrolase, expressed by the AAV products Glybera (24) and Luxturna (25), respectively. It is also possible to generate quantitative, cell-based functional assays, as described for AAV8-UGT1A1 transgene (26). Cell-based assays involving infectivity measurement of AAVs require use of helper viruses like AdV and often specific, immortalized cell lines (e.g., HEK293T) (27).

For ATMPs with transgenes, the correct structure and functionality of the expressed protein is expected to be measured as part of characterisation and non-clinical pharmacodynamic studies (6). Factors impacting biological activity like vector tropism, choice of the promoter, vector infectivity and selectivity of the transgene expression (when using conditional or cell type-specific promoters) need to be addressed as well and taken into account in the product design (6, 27). For the approved AAV products Glybera (24), Luxturna (28), Zolgensma (29), Upstaza (30), Roctavian (31) and Hemgenix (32), publicly available information on such factors is collected into Table 1 from the respective EU assessment reports and literature (33).

**Table 1:** Factors potentially impacting biological activity of AAVs in clinical use.

Product	Vector/origin	Transgene	Promoter	Enhancer	Tissue tropism (based on clinical use) (33)
Glybera	AAV1 (NHP)	hLPL	CMV/IEP	WPRE	CNS, muscle, heart; WPRE increases expression level of hLPL
Luxturna	AAV2 (human)	hRPE65	CMV/CBA	none	Liver, CNS, muscle, eye
Zolgensma	AAV9 (NHP)	hSMN1	CMV/CBA	none	Wide tropism, AAV9 can penetrate the blood brain barrier and drive gene expression throughout the CNS
Upstaza	AAV2 (human)	hAADC	CMV/IEP	HBG2/3	Liver, kidney, retina; truncated HBG2/3 increases expression of the transgene
Roctavian	AAV5 (human)	hFVIII	LSP	none	CNS, lung, eye
Hemgenix	AAV5 (human)	hFIX	LSP	none	CNS, lung, eye

NHP, non-human primate; hLPL, human lipoprotein lipase; hRPE65, human retinal pigment epithelium 65 kDa protein producing gene; hSMN1, human survival motor neuron 1 gene; hAADC, human aromatic L-amino acid decarboxylase gene; hFVIII, human coagulation factor VIII; hFIX, human coagulation factor IX; CMV/EIP, Cytomegalovirus early immediate promoter; CMV/CBA, Cytomegalovirus enhancer/chicken-*β*-actin hybrid promoter; LSP, liver specific promoter; WPRE, Woodchuck hepatitis virus post-transcriptional regulatory element; HBG2/3, human b-globin partial intron 2/partial exon3; CNS, central nervous system.

Potency of oncolytic viruses (OV) is based on their infectivity and capability to lyse the infected cells. OVs are mainly used for oncology indications and often their oncolytic potential and tumor selectivity are enhanced by genetic engineering of the viruses (34). Oncolytic potency may be increased by increasing the replication of the virus or by adding transgenes that express molecules interfering with tumor signaling like TGF *β* (34). Potency assays of OVs include *in vitro* lysis of tumor cells, but also expression of the transgene molecules and their effects like antitumor immune responses in case of Imlygic (HSV with the human granulocyte-macrophage colony-stimulating factor [GM-CSF gene]) (35).

Plain nucleic acids like messenger RNAs are classified as ATMPs only if they are biological, i.e., produced through a biological process like *in vitro* transcription. Such product is aimed to translate *in vivo* a peptide or a protein, which is encoded into the mRNA sequence (36). Thus, also the potency testing is related to the peptide/protein expression and functionality in the target cells, like those of AZD8601 mRNA encoding vascular endothelial growth factor A (VEGF-A) (37). Most of the authorised therapeutic RNA products are mainly synthetic antisense oligonucleotides (not classified as ATMPs) or small interfering RNAs (siRNA), which exert their functions by binding to genes of interest and thus do not require similar functional assays as mRNAs (36).

Genome editing (GE) utilizing bacterial nucleases has been a tool for genome research for many decades, however, a breakthrough in drug development came much later when technology based on clustered regularly interspersed short palindromic repeats (CRISPR) and Cas9-endonuclease was discovered in 2012 (38). Other GE platforms include Zinc Finger Nucleases (ZFN) and transcription activator-like effector nucleases (TALEN). The first approach was *ex vivo* editing of patient CD34+ cells, which when given back to the patient can engraft to the bone marrow and generate a new population of the edited cells providing the therapeutic effect. More recently also *in vivo* gene editing has been tested in clinical trials, where the necessary components (guide RNAs, nuclease or vectors) are administered systemically to the patients. Potency testing for such *in vivo* approach requires analytical methods to verify the precise and efficient cutting activity of the components together in a relevant cell/tissue system, but also follow up of the *in vivo* activity by suitable clinical biomedical measurements. One example of *in vivo* GE is treatment of sickle cell disease (SCD) using the ZNF technology. The approach targets BCL11a gene erythroid-specific enhancer, which deactivates the production of fetal hemoglobin (HbF). Deactivation of the enhancer by *in vivo* GE is expected to restore production of HbF and relief SCD symptoms. For this product the potency testing would mean also testing the HbF levels in the patients and characterizing the quality, expression level and functionality of the protein. The *ex vivo* GE is addressed later in the chapter on genetically modified cells.

### Cell-based ATMPs

Cell-based ATMPs include a wide array of different products from simple isolated, expanded cells (e.g., autologous chondrocytes for cartilage repair) up to highly complex genetically modified cells (e.g., gene edited allogeneic CAR T cells, transduced with a viral vector). For the more simple cell products it is often sufficient to control their potency by measuring cell viability, few specific cell surface markers and their retained, original functionality. For chondrocytes, surrogate markers like Glycosaminoglycans (GAGs), Aggrecan or Collagen Type 2 are often used, but their ability to form proper hyaline cartilage requires *in vivo* or specific *in vitro* methods (39). Such *in vivo* models have included, e.g., human expanded chondrocytes injected into Nude mice (40) and analysis of the implants using hyaline staining. *In vitro* methods include so-called “hanging drop” and pellet cultures, where the chondrocytes form aggregates and further explants that can be analysed for chondrogenic properties and markers (41).

Mesenchymal stem/stromal cells (MSCs) are multipotent cells that can be isolated from blood, bone marrow, adipose tissue or umbilical cord blood. The isolated cells can be differentiated into adipocytes, osteoblasts, and chondrocytes, which have been utilized in treatment of bone and cartilage defects (42, 43). This tri-lineage differentiation assay is often used also as a potency test to demonstrate that the cells have retained their differentiation capacity (43).

In addition, MSCs have immunomodulatory properties, which have led to wide use of these cells for treatment of conditions like Graft versus Host Disease (GvHD) (43). The International Society for Cell and Gene Therapy (ISCT) has published a review of the MSC markers that are used to identify these cells (44), however, it has been later shown that MSCs include different cell populations with different marker profiles and also differing functionalities (44, 45). Furthermore, it has been shown that the age of the donor has big impact on the marker profile, characteristics and functionality of the MSCs (45). The immunomodulatory effects involve expression of cytokines and interaction of the MSCs with host immune cells; the effects are also known to depend on the local microenvironment, where the cells distribute (46). Thus, for MSCs potency testing the cell source and population (proper markers) together with intended MoA (regeneration or immunomodulatory) and clinical indication should be taken into account. The assays measuring immunomodulatory properties depend on whether the cells are aimed for anti-inflammatory or immune-stimulatory use (paracrine effects). MSCs secrete a broad range of bioactive molecules, such as growth factors, cytokines and chemokines, which can be measured for potency (47). The first approved MSC product Alofisel was developed for treatment of anal fistulas (48). The cells are anti-inflammatory, i.e., they suppress proliferation of lymphocytes and inhibit the release of pro-inflammatory cytokines, thereby allowing the tissues around the fistulas to heal. Functional parameters like differentiation capacity, immune-regulation, immune-related proteins and proteins related with regenerative and reparative activity have been listed for Alofisel in the respective public assessment report (48).

Other immunotherapeutic ATMPs include T cells, dendritic cells (DCs) and intact or manipulated natural killer (NK) cells. Potency of modified T-cell products may relate to direct cytotoxicity, secretion of cytokines or proliferative response of recipient peripheral blood mononuclear cells (PBMCs). Methods like ELISpot or flow cytometry (FACS) can be utilized for detection of cytokine-expressing T cells and Mixed Lymphocyte Reaction (MLR) for potency testing of cytotoxic T cells. NK cells can be cytotoxic to infected or transformed cells and due to their functional Fc receptor NK cells play a role in antibody dependent cellular cytotoxicity (ADCC). Potency of NK cells can be measured using assays for cytotoxicity (e.g., target cell lysis by FACS), cytokine production or NK cell proliferation (49).

DCs can present specific tumor antigens to T cells and thus may prove a valuable tool for anti-cancer immunotherapy. Functional potency assay in such case would preferably demonstrate DCs to stimulate antigen-specific T cells, however, alternative potency tests based, e.g., on DC antigen uptake, DC maturation and tumor growth inhibition have been evaluated (50). On the other hand, DCs can also be used to induce tolerance (e.g., peptide loaded DCs). In such case the potency testing would be different, e.g., based on the ability to generate regulatory T cells (Tregs) (51).

Pluripotent stem cells like embryonic stem cells (ESCs) and induced pluripotent stem cells (iPSCs) have capacity to differentiate into any human cell type. They also have an inherent capacity to form teratomas, benign tumors, when being undifferentiated. Therefore, the potency of such cells strongly relates to the intended differentiation status (suitable markers and proliferation) and their intended MoA in each indication. Both ESCs and iPSCs have been used to treat retinal diseases like retinitis pigmentosa (RP) or age-related macular degeneration (AMD) (52). For such use it is critical to control the differentiation of the pluripotent cells into photoreceptor or retinal pigment epithelium (RPE) cells; potency testing in this case would combine differentiation assays/markers with methods assessing target cell type structure and functionality. Limbal stem cells, isolated from patients´ own cornea, have been utilized in Holoclar, which was approved for the treatment of moderate to severe limbal stem cell deficiency (53).

Genetically modified cells include wide variety of products for different indications, like CAR T cells or T-cells with modified T-cell receptor (TCRs), CAR NK cells and genetically modified CD34+ hematopoietic stem cells intended for *ex vivo* gene therapy. In addition, gene editing technologies have made it possible to modify in principle any cell type for therapeutic use. Autologous CAR T cells have been in the center of attention since the approval of first anti- CD 19 CAR T products Kymriah (54) and Yescarta (55) in 2018. Potency release tests described in the European public assessment reports (EPARs) include combination of cell viability, anti-CD19 CAR expression and T-cell activation (cytokine release), however, transduction efficiency (TE) and vector copy number (VCN) play also a critical role in the final activity of the cell population and are expected to be part of the release testing. CAR expression is usually measured using FACS, whereas TE and VCN require assays detecting the viral vector in the cells, usually using PCR-based methods. Furthermore, functionality/potency of the active substance has been addressed through characterisation studies, where parameters like CAR expression, antigen recognition and engagement, T-cell activation/release of cytokines, killing of target cells, composition and phenotypes of the T-cells and multiplicity of infection have been measured (54, 55).

Although the anti- CD19 CAR T products have shown outstanding results in treatment of lymphomas and leukemias, there are still patients that do not benefit from the therapy. One reason often raised is the poor health status of the T-cells isolated for manufacturing from patients, who have been through multiple prior treatments, e.g., with cytotoxic cancer drugs. More recently it has been recognized that also the design of the CAR construct, the cell composition and the components of the viral vectors used may have significant impact on the potency of the product (56, 57). CAR binding affinity and its expression level define the antigen-binding properties of the receptor and thus the efficacy of target cell recognition, which is critical for binding and elimination of the tumor cells. In addition, the co-stimulatory domains are required for full activation of the T-cells and the other components, like hinge and transmembrane domain, play important roles for optimal structure of the chimeric receptor; thus characterisation of the product as a whole for optimal functionality/potency is essential and requires multiple analytical tools.

*Ex vivo* gene therapy using autologous CD34+ cells with integrating lenti-or gamma-retroviral vectors has become a significant option especially for treatment of rare, inherited diseases, where long term expression of the target protein is required. The first approved genetically modified CD34+ cell-based products include Strimvelis (58), Zynteglo (59), and Libmeldy (60), approved in 2016, 2019, and 2020, respectively. For such products the potency testing usually includes cell viability and expression of the transgene protein, but also % vector positive cells, vector copy number and transduction efficiency. However, functional potency of transduced CD34+ cells may be difficult to address *in vitro* after manufacturing, as part of the biological activity takes place only after administration. CD34+ cells are hematopoietic stem cells, which are expected to find their way back to the bone marrow and engraft. In the bone marrow the cells proliferate and differentiate into different hematopoietic cell lineages, expressing the intended protein. Thus, true functional potency of transduced CD34+ cells can only be measured from clinical samples of the treated patients using bioanalytical tools to measure engraftment capacity (e.g., colony formation assay), differentiation into various cell lineages (FACS) and expression level/quality of the transgene product. Sometimes the cells have to differentiate into specific cell types *in vivo* before they can deliver the therapeutic effect, like in case of Wiskott Aldrich Syndrome (WAS), which requires restoration of functional lymphocytes and platelets to reduce the immunological symptoms and bleeding (61).

Genome editing (GE) provides tools to remove or modify existing genes/sequences in cells and to add new genes/sequences into the genome of the cells; both *in vivo* and *ex vivo* approaches are used. In many *ex vivo* cases the editing is part of manufacturing, used together with other gene therapy tools like viral vectors. Use of GE requires that all added/removed/modified cell characteristics are controlled; if those have impact on potency, it should be considered in the potency testing scheme.

## Limitations/challenges of potency assays

Potency testing has been found to be one of the most challenging aspects of the CMC development for ATMPs. Concerns around potency have resulted in numerous major objections during the review of the MAAs, which in worst cases have resulted into withdrawal or rejection of the application (21). One critical issue for the potency testing of ATMPs is that the biological activity can be mediated by numerous factors and thus one single marker or assay may not fully reflect the functionality of the product. On the other hand, analytical methods have to be validated according to ICH guidance in case those are used for release or stability testing or as in process controls (8). Methods, that are used for characterisation purposes do not need to be fully validated but qualified to ensure reliable and repeatable results (using suitable controls). Functional potency assays of ATMPs are often difficult to validate, due to the cellular components and inherent variability caused by the biological materials and the manufacturing processes. For autologous products often the number of cells available is limited, which hampers wide testing at release and may require to follow simple markers or surrogate assays for potency. In the EU this is acceptable, as long as there is a functional potency assay for characterisation purposes and the results of the potency assays correlate with each other (5). For autologous products intended to be given fresh, the limited shelf life before patient administration may hamper potency testing in case it takes several hours before the result is obtained. The use of surrogate markers could enable release and if a biological assay is still needed, results could be provided after administration, provided this is accepted by the authorities. Impurities may have a negative impact on potency, e.g., in case of AAVs that contain a lot of empty or partially packed particles that can compete for receptor binding sites in target cells and/or induce immune responses against the vector (62).

In some cases, relative potency assays are used, where the samples are compared to an existing reference standard. This is acceptable as long as the validity and stability of the reference material is ensured. However, such an approach may bring challenges regarding the statistical assessment of the results and especially concerning parallelism of the dose–response curves (9). An additional challenge for relative potency testing could be the (re-)qualification and comparability of further reference standard materials when the first lot is exhausted. In the worst case, the assay may need complete revalidation if it is not possible to generate a comparable reference standard. One important issue to keep in mind when selecting potency assays is the impact of the process and of the finished product formulation on the performance of analytical methods. Some reagents and/or process steps may inhibit or enhance the analytical capability, like DNAse treatment that may be used for AAVs before measuring the viral titre (63). On the other hand, when AAVs are stored in high concentration or non-optimally formulated, this may lead to aggregation, which is known to hamper analytical measurements of AAV products (63, 64).

## Non-clinical models to support potency testing

Given the complexity and diversity of ATMPs, development of a suitable and relevant non-clinical testing program can be difficult and needs to be tailored according to the specificities of each ATMP. For all medicinal products, in non-clinical development, it is a requirement to show evidence that products have therapeutic activity (12, 13). In essence, the developer needs to generate data demonstrating a potential therapeutic effect that could be achieved in clinical use (proof of concept, PoC). Normally such data is generated in various *in vitro* or, more commonly, *in vivo* disease models, often dominated by models in rodents (mice and rats). The effect inflicted on the disease in such models should be solely dependent on the pharmacodynamic (PD) MoA of the investigational medicinal product (IMP) on its target. For non-ATMPs this mostly involves agonistic/antagonistic molecular binding-effects on the dedicated target or, for biotechnological IMPs, binding of recombinant proteins to the devoted receptor. For small chemicals, the pharmacodynamic effect are mostly non-species specific, or at least activity against the same target in various species can be compared and any differences can be accurately taken into account. For biological products the PD effects are more commonly human-specific simply due to the protein nature of these IMPs. Thus, sometimes *in vivo* pharmacodynamic proof-of-concept (PoC)/MoA data is generated with the homologous product complemented with *in vitro* data depicting any potency differences between the species. Alternatively, the human product (e.g., protein) is conserved between humans and the species used in the non-clinical disease model and thus no *in vitro* comparison is generally needed.

The pharmacodynamics of the CGTs/ATMPs, especially cell-based CGTs/ATMPs are far more complex than other pharmaceuticals. Consequently, the effort of generating pharmacodynamically relevant data with the human IMP in animals is much more challenging. For instance, the complexity of a human cell-based IMP and its interaction with other cells in a xenogeneic host can be fundamentally different compared to the same cell–cell interaction in the allogenic or autologous human host. Such differences do often affect the actual *in vivo* PD/PoC data, which in turn creates uncertainties when extrapolating PD activity from animals to human.

The issues described above do not affect vector-based ATMPs to the same extent. However, it is vital that the pharmacodynamic effect of the transgene is measurable in the selected non-clinical species. Also, species differences relating to viral tropism can have a major impact on the data generated in the non-clinical test species, especially when targeting specific structures within the body or when the capsid has been modified to enhance transduction of certain human cells. This may result in substantial differences in the transduction efficiency between human and non-human species which in turn will affect the level of the expressed transgene and thereby its PD effect, resulting in differences in potency between species. These shortcomings have been counteracted by many developers by generating data with homologous products. However, such models are always questioned by regulators for relevance and developers normally need to present extensive PD data bridging the homologous animal product to the intended human IMP.

The non-clinical *in vivo* pharmacodynamic effect presented by the developer during early development is also a vital part of early CMC development. This is especially true for establishing a relevant potency assay for the product. Thus, any inconsistencies or matters of irrelevance in the non-clinical *in vivo* PoC models, and the generated PD data in such models, will have a substantial impact on the potency assay of the IMP, which in turn could compromise the reliability of the potency data when continuing from non-clinical to clinical development. Considerations and advice for the non-clinical PD studies for ATMPs can be found both from the EU/EMA (5–7) and the US/FDA (15, 65), guidelines.

## Correlation of potency with clinical efficacy

From a regulatory perspective demonstrating a positive benefit–risk balance should be the goal of any clinical development program. The clinical efficacy and safety are among others closely linked to the potency at the sites of pharmacological activity. This holds for any pharmaceutical, including ATMPs. Clinical studies are generally relatively insensitive to detect minor differences in potency, most often due to lack of highly sensitive clinical endpoints and especially the inter-subject variability. However, a slight drift in potency – either during the development or post-authorization – may have limited clinical detectable impact on the average patient but could lead to lack of effect and/or unexpected adverse events in subgroups of highly sensitive patients. Proper potency testing and reassurance of consistency throughout the clinical development and into the commercial production is therefore a mandatory regulatory requirement (12, 13). Although bridging between different versions of the product used in early-stage clinical trials can be done by appropriate analytical comparability and non-clinical testing (11) it is a general regulatory expectation that pivotal clinical studies are carried out using ATMPs representative of the intended commercial product. These regulatory principles get challenged by highly individualized ATMPs such as those based on *ex-vivo* manipulated autologous material. In such cases regulators may challenge the suitability of analytical comparability and non-clinical tests in establishing potency and comparability toward the final product. This will in some cases lead to a request to provide for assessment the clinical data from individual patient data collected throughout the development to support establishing the product potency. For Zolgensma the comparability between early and late manufacturing process could not be assured by analytical testing and the benefit/risk conclusion had to be based solely on the available later clinical data (29). The lack of established comparability between early and commercial batches together with the limited clinical efficacy and safety data resulted in a conditional approval, which was granted with commitments to collect additional clinical data and to further study correlation of the critical quality attributes (genomic titre, infectious titre, *in vitro* relative potency) and clinical outcome.

In case the data for both clinical efficacy and the potency testing raise concerns, there is a high risk of failure at the MAA/BLA (US Biologics License Application) phase. For Holoclar this situation was noted, but as the benefit risk balance was considered positive for high unmet medical need, a conditional marketing authorization was granted with commitments to conduct an additional prospective clinical study and to explore additional potency markers post-approval (53).

As stated above clinical endpoints will in many circumstances be too insensitive to pick up minor differences in potency so selecting the most sensitive endpoint will be important. Sponsors will in most such cases benefit from seeking scientific advice from regulatory authorities to agree on endpoints for clinical potency/efficacy assessment.

## Discussion

As described above, development of quantitative, relevant, fast potency assays for cell and gene therapy products can be challenging and time consuming. Lack of robust potency assay(s) can in worst case hamper control of the product quality at release and during storage, validation of the manufacturing process and comparability testing, in case of process changes. Thus, the strategy for potency testing together with assay development should be considered early on, preferably before pivotal non-clinical studies to support the translation towards the clinical studies.

Cell and gene therapy products are getting more and more complicated, as, e.g., genome editing technologies are advancing. The new techniques and the use of viral vectors will change the normal characteristics of the cells, often having impact also on their functionality. For example, high overexpression of proteins from transgenes or removal of multiple functional genes may impact normal cell homeostasis/functionality and lead to consequences that may become visible only with *in vivo* studies. Therefore, it is critical that the strategy for potency testing takes into consideration the MoA, relevance of the non-clinical models and possibility to establish correlation with clinical efficacy. In most complex cases one single potency assay may not be sufficient to cover all functional aspects of the product. Furthermore, it would be good to consider quantitation and validation aspects of potency assays early on and develop complementary assays for release and characterisation purposes, where difficulties are anticipated. Whenever challenges in potency testing are observed, early scientific advice from regulatory authorities is recommended.

## Author contributions

All authors listed have made a substantial, direct, and intellectual contribution to the work and approved it for publication.

## Conflict of interest

PS, BC, CK, and TR were employed by company NDA Group.

The remaining authors declare that the research was conducted in the absence of any commercial or financial relationships that could be construed as a potential conflict of interest.

## Publisher’s note

All claims expressed in this article are solely those of the authors and do not necessarily represent those of their affiliated organizations, or those of the publisher, the editors and the reviewers. Any product that may be evaluated in this article, or claim that may be made by its manufacturer, is not guaranteed or endorsed by the publisher.

## Author disclaimer

The views expressed in this article are the personal views of the author(s) and may not be understood or quoted as being made on behalf of or reflecting the position of the regulatory agency/agencies or organizations with which the author(s) is/are employed/affiliated. The manuscript has been subjected to internal peer review at EMA.
